# Effect of Neonatal Exposure to Poly(Ethylene Glycol)-*block*-Poly(Lactic Acid) Nanoparticles on Oxidative State in Infantile and Adult Female Rats

**DOI:** 10.1155/2017/7430435

**Published:** 2017-09-10

**Authors:** Monika Dvořáková, Eva Rollerová, Soňa Scsuková, Alžbeta Bujňáková Mlynarčíková, Lucia Laubertová, Ingrid Žitňanová

**Affiliations:** ^1^Department of Medical Chemistry, Biochemistry and Clinical Biochemistry, Faculty of Medicine, Comenius University, Sasinkova 2, 813 72 Bratislava, Slovakia; ^2^Faculty of Public Health, Department of Toxicology, Slovak Medical University, Limbová 12, 833 03 Bratislava, Slovakia; ^3^Institute of Experimental Endocrinology, Biomedical Research Center, Slovak Academy of Sciences, Dúbravská cesta 9, 845 05 Bratislava, Slovakia

## Abstract

Our goal was to evaluate the potential health risk of the polymeric NP, poly(ethylene glycol)-*block*-poly(lactic acid) (PEG-*b*-PLA), from the view of redox imbalance of the organism in two different life stages. Female Wistar rats were neonatally administered intraperitoneally with PEG-*b*-PLA NPs [20 mg/kg of b.w. (PEG20) or 40 (PEG40) mg/kg of b.w.] from postnatal day 4 (PND4) to PND7. We measured antioxidant capacity (TEAC), level of protein carbonyls and lipoperoxides in plasma, activities of catalase, glutathione peroxidase (GPx), and superoxide dismutase (SOD) in hemolysates of infantile (sacrificed on PND17) and adult (sacrificed after PND176) rats. Compared to controls, neonatal PEG40 exposure induced a significant TEAC reduction in the infantile rats. Protein carbonyls and lipoperoxide levels were not affected after any dose of PEG-*b*-PLA NP administration. In adult rats, PEG20 administration caused a significant decrease of protein carbonyl levels compared to controls. In infantile rats, both doses of PEG-*b*-PLA NP administration increased catalase, Gpx, and SOD activities compared to controls. Surprisingly, in adult rats, the activities of Gpx and SOD decreased significantly after administration of both doses of PEG-*b*-PLA NPs. Obtained data indicate a possible age-related association between the oxidative status and neonatal PEG-*b*-PLA NP administration in female rats.

## 1. Introduction

In recent years, nanomaterials have been studied in detail due to irreplaceable properties of nanoparticles (NPs) (their size less than 100 nm, shape and surface, chemical composition, and physicochemical properties). Over the past decade, increasing interest in nanotechnology has been recorded with focus on the field of medicine involving prevention, diagnostics, and treatment of human diseases such as cancer [1–5].

Recently, attention has been focused on nanoparticle pharmaceutical applications, especially on the delivery of drugs, proteins, and DNA to special targets in the body. Nanoparticles are able to protect drugs from degradation in the gastrointestinal tract, as well as to carry slightly water-soluble drugs. In addition, they can remain in the blood circulation for a long time and release the drug in controlled intervals [6] and thus enhance its cytotoxic effects and reduce nonspecific targeting of healthy cells.

Polymeric NPs and polymeric micelles are formed from various biocompatible and biodegradable polymers [poly(lactic acid)—PLA acid, poly(lactic-co-glycolic acid)—PLGA, polyethylene glycol—PEG]. They consist of a hydrophobic core containing the drug and of a shell able to stabilize the NPs in fluid state and to interact with the solvent. They improve the effectivity and safety of transferred drugs. Their surface can be modified by vehicles, for example, detergents, which provide encapsulation of drugs, their protection against degradation and also enable the transport and release of active substances through the blood-brain barrier (BBB) without their damage [7] and act as carriers for drug delivery in cancer therapy [5]. Moreover, modification of NP's surface by L-glutamic acid, polysaccharides (chitosan), or polymer coatings (PEG) is often used to reduce the cytotoxicity associated with NPs [8].

For poorly water-soluble drugs, the polymeric NP poly(ethylene glycol)-*block*-poly(lactic acid) (PEG-*b*-PLA), an amphiphilic block copolymer (ABC), has been designed as a drug carrier. The PEG backbone is responsible for the hydrophilicity of the NP, while the hydrolytically labile PLA linkages provide biodegradability. The polymerizable end groups provide the ability to form a cross-linked network [9]. It has been proven that PEG-*b*-PLA NPs are able to pass the BBB, and due to this skill, they are one of the most favorable drug carriers for the CNS [10–13].

Despite the increased popularity of NPs in the field of nanomedicine, their use is often limited due to their potential toxicity and long-term side effects [3, 14, 15]. Especially, the size of the nanoparticle is considered the key factor of its toxicity. In general, the smaller the nanoparticle, the higher cytotoxicity it exhibits. In addition, the structure and shape could be crucial for the toxicity [15–17]. It has been suggested that cytotoxic effects are induced by reactive oxygen species (ROS) formation resulting in damage to biologically important molecules, for example, nucleic acids, lipids, and proteins and finally leading to cell death [3, 5, 17–21]. Moreover, in various organs, many NPs have been shown to be toxic through their accumulation and upregulation of proinflammatory genes and production of ROS [18–21].

To broad the safety profile of PEGylated PLA NPs (copolymer PEG-*b*-PLA [CH_3_O(CH_2_CH_2_O)_x_(COCHCH_3_O)_y_H]), we have studied neuroendocrine and developmental toxicity induced by PEG-*b*-PLA NPs in female Wistar rats after their neonatal exposure. Our study has demonstrated the adverse effects of short-time neonatal/developmental exposure to PEG-*b*-PLA NPs on somatic and pubertal benchmarks and some endpoints of reproductive functions (estrous cyclicity) in the female rats. There are also indications that PEG-*b*-PLA NPs might interfere with the activation and function of the hypothalamic-pituitary-gonadal (HPG) axis. Hormonal effects induced by PEG-*b*-PLA NPs might play an important role in the nanoreprotoxicity of PEG-*b*-PLA NPs at both the central neuroendocrine and gonadal levels (progesterone production) [22].

Moreover, neuroendocrine disrupting effect of neonatal exposure of female rats to PEG-*b*-PLA NPs has been confirmed by examining ex vivo LHRH-induced LH release from gonadotropic cells isolated from infantile (PND15) and adult (PND176) animals. The significant increase in the pituitary LH secretion by the action of PEG-*b*-PLA NPs has persisted from the infantile to adult life period [23].

Polyethylene glycol coating reduces the toxicity of NPs in the cells [8]. However, according to our knowledge, there is a lack of studies monitoring the relationship between the PEG-*b*-PLA administration and oxidative state of the organism. Therefore, the aim of our study was to examine effects of neonatal exposure to PEG-*b*-PLA NPs on selected parameters of redox imbalance in two life stages of female rats, infantile and adult.

## 2. Material and Methods

### 2.1. Preparation of Suspension of NPs and Its Characterization

Micelles of PEG-*b*-PLA were prepared freshly by the modified method of Du et al. [24] and Shin et al. [25].

Briefly, copolymer poly(ethylene glycol)-*block*-poly(lactic acid) (PEG-*b*-PLA) [CH_3_O(CH_2_CH_2_O)_x_(COCHCH_3_O)_y_H, PEG with average Mn = 350 g/mol, PLA with average Mn = 1000 g/mol, CAS 9004-74-4, Sigma-Aldrich, Steinheim, Germany] (20 mg) was dissolved in 2 ml of tetrahydrofuran (THF; anhydrous, inhibitor free, purity ≥99.9%; Sigma-Aldrich, Steinheim, Germany). Under moderate stirring, 10 ml of the ultrapurified water (Millipore Milli-Q Synthesis, 18.5 mol/l) was added dropwise. Two hours later, after evaporation of THF (mild vacuum, 1 h, 48°C), polymeric micelles were obtained. Final suspension of PEG-*b*-PLA was diluted with water to the concentration of 20 mg/10 ml. Before injection, PEG-*b*-PLA suspension was vortexed for 1 min.

Suspension of PEG-*b*-PLA was characterized by the transmission electron micrography (TEM), electrophoretic light scattering (ELS), and dynamic light scattering (DLS) methods.

Physical particle size, general state of agglomeration/aggregation, and morphology were determined by TEM using a transmission electron microscope JEM 1200 (JOEL, Tokyo, Japan) with 120 kV voltage. Size distribution of PEG-*b*-PLA was evaluated by DLS with a NICOMP™ 380 ZLS Particle Sizer (Santa Barbara, CA, USA). Size measurement was performed at 25°C and a scattering angle of 90°. The employed NICOMP software can automatically recognize up to three size distributions of particles concurrently present through a patented software algorithm. Zeta potential was measured by Nicomp Submicron Particle Sizer Autodilute Model 380 (Santa Barbara, CA, USA) using the ELS method.

TEM demonstrated spherical shapes of NPs, and the average primary particle size (PPS) suspended in deionized water was about 50 nm (Table 1). Zeta potential value, measured in triplicate at pH 7.0, was 28.73 ± 1.44 mV (Table 1). Micelle dispersion resulted in size distribution with two main peaks of secondary particle sizes (SPS) averaged 64.9 ± 10.5 nm and 911.4 ± 177.6 nm (Table 1), which indicates that the PEG-*b*-PLA micelles were aggregated in solution. Similar to the findings by Shin et al. [25], PEG-*b*-PLA micelles were stable for 24 h at ambient temperature as monitored by TEM. More details are presented in the paper by Rollerova et al. [22].

### 2.2. Study Design [22]

Briefly, in our study, 64 female specific-pathogen-free Wistar rats were included. To simulate intravenous administration of PEG-*b*-PLA micelles to humans, intraperitoneal administration was used. Daily, on postnatal days (PND) 4–7, neonatal rats were intraperitoneally injected with one of two doses of PEG-*b*-PLA NP either 20 mg/kg (infantile female rats, *n* = 10; adult female rats, *n* = 12) or 40 mg/kg of b.w., respectively, (infantile female rats, *n* = 10; adult female rats, *n* = 12). In negative control groups (infantile female rats, *n* = 10; adult female rats, *n* = 10), animals were injected with ultrapurified water after evaporation of tetrahydrofuran (vehicle used in NP preparation). Female rats with neonatally administered NP were sacrificed by decapitation at two different life stages: infantile and adult. Infantile female rats were sacrificed on PND17 and adult animals on the day of the first estrus after PND176. For sacrification, ketamine/xylazine anesthesia (60/10 mg/kg of b.w.) [ketamine (Narketan) Vetoquinol Ltd., Czech Republic; xylazine (Xylariem) Riemser Arzneimittel AG, Germany] was used.

The State Veterinary and Food Administration in the Slovak Republic has approved the protocol of our study. Standard Operating Procedures—Good Laboratory Practice (GLP)— of the Department of Toxicology, Slovak Medical University, Bratislava, in compliance with the European Convention for the Protection of Vertebrate Animals used for Experimental and other Scientific Purposes (ETS 123) were applied to the animal care. The study meets the WHO International Ethical Guidelines for Biomedical Research involving experimental animals, and it complies with the Slovak Statutory Orders Number 377/2012 Z. z. and Number 436/2012 Z. z. (Collection of Laws).

### 2.3. Plasma and Hemolysate

After decapitation, blood samples were collected into commercial tubes with Li-Heparin as an anticoagulant. Samples were centrifuged (1200 ×g, 5 min, 4°C) and plasma was aliquoted, shock frozen, and stored at −80°C until further analysis. Blood plasma aliquots were used for determination of parameters of oxidative stress (antioxidant capacity, protein carbonyls, and lipoperoxides).

After washing (0.5 ml) with 5 ml of physiological solution (three times), the erythrocyte suspension was centrifuged (660 ×g, 5 min, 4°C) and hemolyzed in chilled distilled water.

Hemolysates were aliquoted, frozen, and stored at −80°C. They were used for determination of hemoglobin concentration and activities of antioxidant enzymes (glutathione peroxidase, catalase, and superoxide dismutase).

### 2.4. Trolox Equivalent Antioxidant Capacity

Trolox equivalent antioxidant capacity (TEAC) in plasma was analyzed spectrophotometrically according to Re et al. [26]. The TEAC values are expressed in mmol of trolox/l of plasma using trolox (hydrophilic form of vitamin E) as a standard.

### 2.5. Protein Carbonyls

Concentration of protein carbonyls is related to the amount of proteins present in plasma. The concentration of proteins was measured by Pierce™ BCA Protein Assay Kit (Thermo Scientific, USA).

To determine protein carbonyl levels in the rat plasma, the commercial kit OxiSelect™ Protein Carbonyl ELISA Kit (Cell Biolabs, Inc., USA) was used. Protein carbonyl levels are expressed as nmol of carbonyls/mg of proteins.

### 2.6. Lipoperoxides

The level of lipid peroxides in plasma was measured spectrophotometrically according to the method by El-Saadani et al. [27]. Lipoperoxide levels are shown in nmol/ml of plasma.

### 2.7. Catalase Activity

To determine the catalase activity in lysates of erythrocytes, the method according to Bergmeyer [28] was used. The activity was expressed in *μ*kat/g hemoglobin (Hb). In the hemolysates, hemoglobin concentration was determined using the Drabkin's reagent [29].

### 2.8. Glutathione Peroxidase Activity

To determine glutathione peroxidase (GPx) activity in lysates of erythrocytes, the commercial Glutathione Peroxidase Assay Kit (Cayman Chemical, USA) was used. Activity of the enzyme is expressed as nU/ml/mg Hb.

### 2.9. Superoxide Dismutase Activity

Superoxide dismutase activity was evaluated in lysates of erythrocytes by SOD Assay Kit (Sigma-Aldrich Co., USA). Activity of the enzyme was expressed in U/mg Hb, where 1 U of SOD activity is defined as the amount of enzyme able to inhibit the rate of chromagen reduction by 50%.

### 2.10. Statistical Analysis

The statistical analyses were performed using Stats Direct 3 Statistical software, version 2.3.7. (StatsDirect® Ltd., UK). Significance level was set at *p* < 0.05. Due to the not normally distributed data, the median with interquartile range (IQR) with minimal and maximal values was used. To detect differences between individual groups, Mann–Whitney *U* test was used for particular variables. The associations between parameters were analyzed with Spearman's correlations. Graphical representations of data were done using box-and-whisker plot. Charts of correlations were made using Spearman's rank correlation.

## 3. Results

### 3.1. Trolox Equivalent Antioxidant Capacity (TEAC)

We have not observed any significant age-related changes (*p* > 0.05) of TEAC in vehicle-treated female rats (controls) (Table 2).

**Figure 1:**
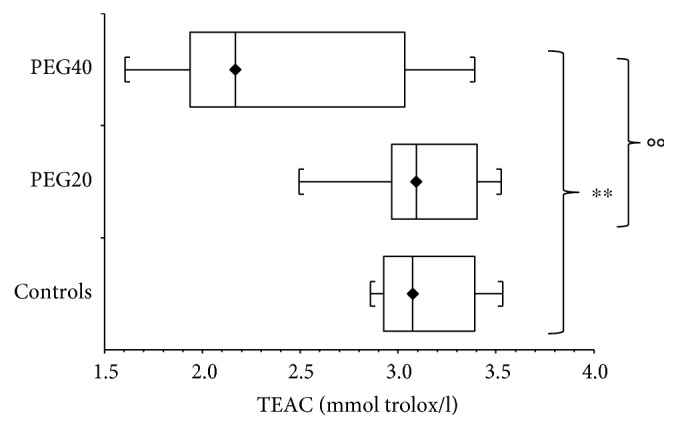
Plasma antioxidant capacity (TEAC) of infantile female rats after neonatal PEG-*b*-PLA administration (PEG20-dose 20 mg/kg of b.w. or PEG40–40 mg/kg of b.w.). Values are listed as median with interquartile range (Q1-Q3, 25–75%). ^∗∗^*p* < 0.01: Controls versus PEG40. °°*p* < 0.01: PEG20 versus PEG40.

In infantile rats (PND17), neonatal administration of PEG20 induced no significant changes (*p* > 0.05) of TEAC when compared with the corresponding control group. Neonatal administration of PEG40 resulted in significant (*p* < 0.01) TEAC reduction compared to controls. Similarly, the significant difference was found between PEG20- and PEG40-treated groups (*p* < 0.01) (Figure 1).

In adult rats (PND176), after neonatal administration of either PEG20 or PEG40, no significant changes (*p* > 0.05) in TEAC were found when compared to the corresponding control group.

### 3.2. Protein Carbonyls

No significant age-related changes (*p* > 0.05) in protein carbonyl levels were observed in vehicle-treated female rats (controls) (Table 2).

Compared to controls, no significant changes (*p* > 0.05) in protein carbonyl levels were observed in infantile rats neonatally injected with either PEG20 or PEG40.

**Figure 2:**
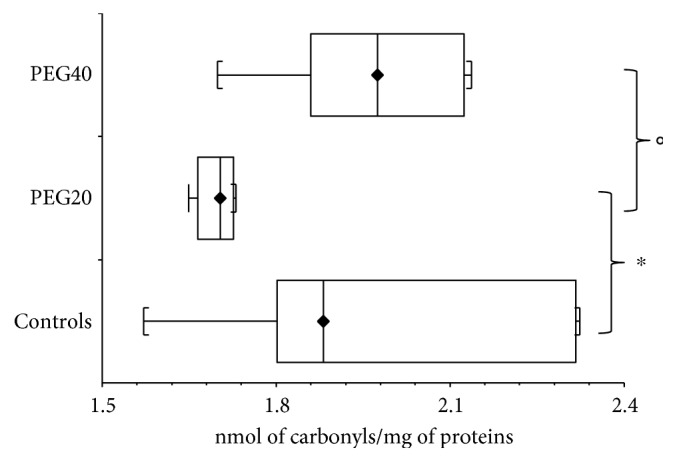
Protein carbonyls concentration in plasma of adult female rats after neonatal PEG-*b*-PLA administration (PEG20-dose 20 mg/kg of b.w. or PEG40–40 mg/kg of b.w). Values are listed as median with interquartile range (Q1-Q3, 25–75%). ^∗^*p* < 0.05: Controls versus PEG20. °*p* < 0.05: PEG20 versus PEG40.

On the contrary, in adult rats, protein carbonyl levels were significantly decreased (*p* < 0.05) after neonatal PEG20 administration compared to the corresponding control and PEG40 groups (Figure 2). Concentration of protein carbonyls after neonatal PEG40 exposure was not changed (*p* > 0.05) when compared with controls in adult animals.

### 3.3. Lipoperoxides

In contrast to TEAC and protein carbonyls, significant decrease (*p* < 0.01) in lipoperoxide levels was observed in adult control females compared to infantile control female rats (Table 2). In the infantile rats, neonatal administration of either PEG20 or PEG40 caused no significant changes (*p* > 0.05) in lipoperoxide levels compared to corresponding controls.

Similarly, in adult rats, both doses of neonatally administered PEG-*b*-PLA NPs caused no significant changes (*p* > 0.05) of lipoperoxide levels compared to the corresponding control group.

### 3.4. Catalase Activity

**Figure 3:**
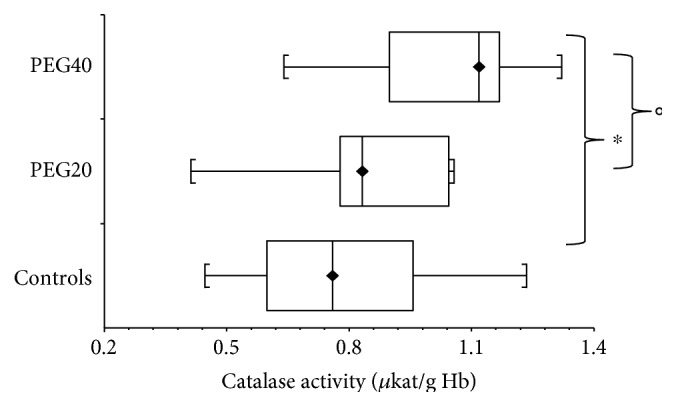
Activity of catalase in hemolysates of erythrocytes of infantile female rats after neonatal PEG-*b*-PLA administration (PEG20-dose 20 mg/kg of b.w. or PEG40–40 mg/kg of b.w.) Values are listed as median with interquartile range (Q1-Q3, 25–75%). ^∗^*p* < 0.05: Controls versus PEG40. °*p* < 0.05: PEG20 versus PEG40.

Compared to controls, the increase of catalase activity in hemolysates of infantile rats was measured after PEG20 neonatal administration (Table 2). The significant difference was found also between the PEG20 and PEG40 groups of female rats (*p* < 0.05) (Figure 3).

Compared to controls, we found no significant changes (*p* > 0.05) in catalase activity in adult rats either in PEG20 or PEG40-treated groups (Table 2).

### 3.5. Glutathione Peroxidase Activity

Glutathione peroxidase (GPx) activity determined in hemolysates of erythrocytes was significantly increased (*p* < 0.001) in adult control female rats compared to infantile controls (Table 2).

**Figure 4:**
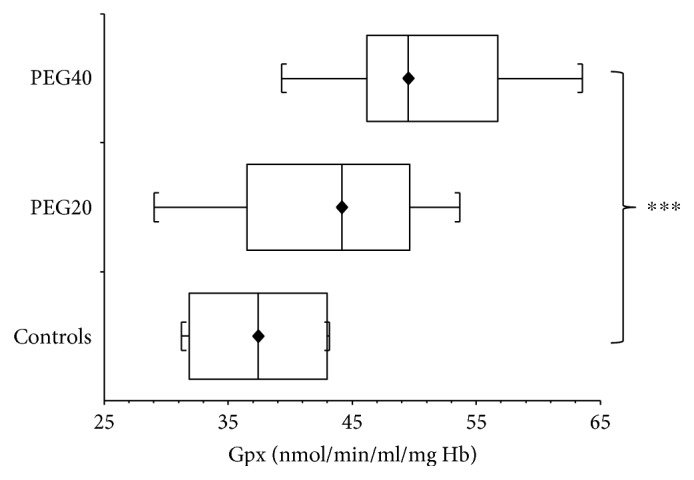
Glutathione peroxidase activity (GPx) in hemolysates of erythrocytes of infantile female rats after neonatal PEG-*b*-PLA administration (PEG20-dose 20 mg/kg of b.w. or PEG40–40 mg/kg of b.w.). Values are listed as median with interquartile range (Q1-Q3, 25–75%). ^∗∗∗^*p* < 0.001 - Controls versus PEG40.

In infantile rats, GPx activity was elevated (*p* > 0.05) after neonatal PEG20 administration compared to controls. The significant increase of GPx activity (*p* < 0.001) was detected after neonatal PEG40 administration (Figure 4).

**Figure 5:**
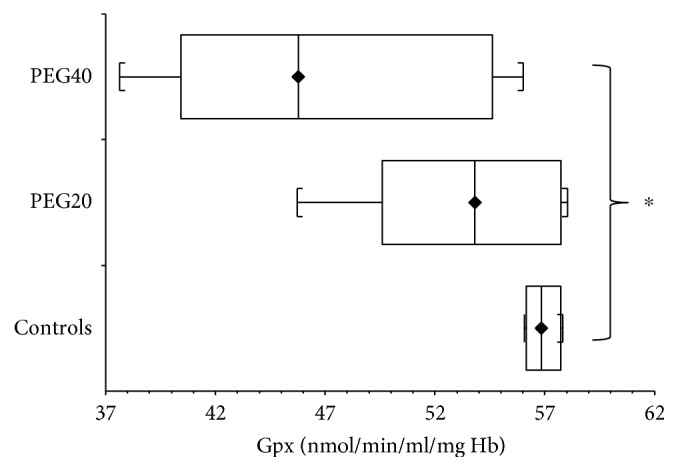
Activity of glutathione peroxidase (GPx) of hemolysates of erythrocytes of adult female rats after neonatal PEG-*b*-PLA administration (PEG20-dose 20 mg/kg of b.w. or PEG40–40 mg/kg of b.w.). Values are listed as median with interquartile range (Q1-Q3, 25–75%). ^∗^*p* < 0.05: Controls versus PEG40.

On the contrary, in adult rats, the GPx activity was decreased (*p* > 0.05) after PEG20 neonatal administration compared to corresponding controls. The significant reduction (*p* < 0.05) of GPx activity was detected after neonatal PEG40 administration (Figure 5).

### 3.6. Superoxide Dismutase Activity

Superoxide dismutase (SOD) activity in hemolysates of erythrocytes was increased in adult control female rats compared to infantile control animals (*p* < 0.05) (Table 2).

**Figure 6:**
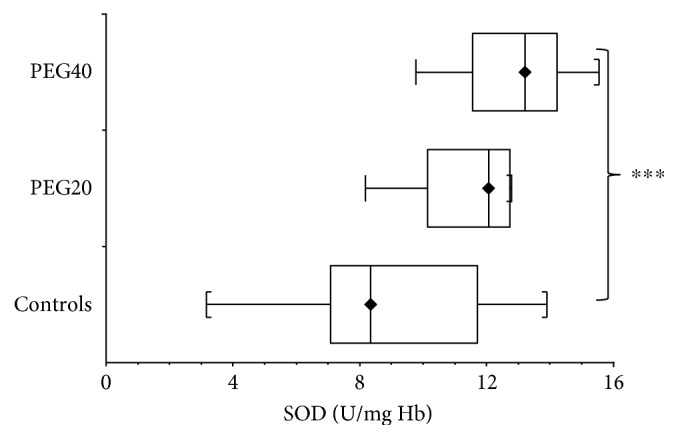
Superoxide dismutase (SOD) activity in hemolysates of erythrocytes of infantile female rats neonatal after PEG-*b*-PLA administration (PEG20-dose 20 mg/kg of b.w. or PEG40–40 mg/kg of b.w.). Values are listed as median with interquartile range (Q1-Q3, 25–75%). ^∗∗∗^*p* < 0.001: Controls versus PEG40.

In infantile rats, SOD activity was elevated (*p* > 0.05) after neonatal PEG20 administration compared to controls. The significant increase of SOD activity (*p* < 0.001) was detected after neonatal PEG40 exposure (Figure 6).

**Figure 7:**
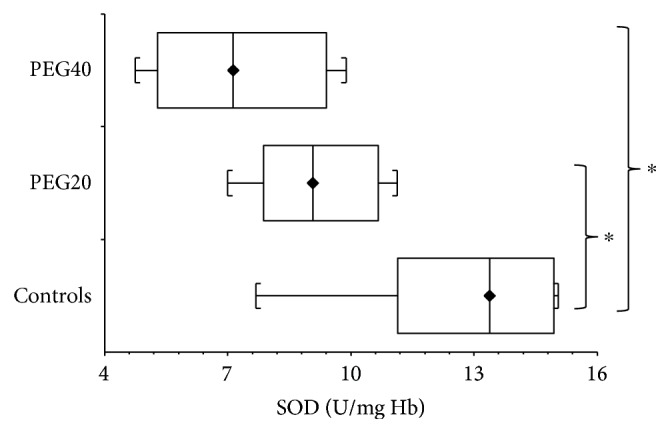
Superoxide dismutase (SOD) activity in hemolysates of erythrocytes of adult female rats after neonatal PEG-*b*-PLA administration (PEG20-dose 20 mg/kg of b.w. or PEG40–40 mg/kg of b.w.). Values are listed as median with interquartile range (Q1-Q3, 25–75%). ^∗^*p* < 0.05: Controls versusPEG20 or PEG40.

Remarkably, in adult rats, SOD activity after PEG-*b*-NP exposure had an opposite trend. When compared to controls, neonatal PEG20 administration caused a significant decrease (*p* < 0.05) in SOD activity, and this decline was even more pronounced (*p* < 0.05) in the PEG40 group (Figure 7).

### 3.7. Correlations

Significant positive and negative correlations found between measured parameters in both life stages of rats (infantile and adult animals) administered with PEG20, PEG40, or deionized water (controls) are reported in Table 3.

## 4. Discussion

The aim of the present study was to investigate effects of neonatal PEG-*b*-PLA NP administration to female rats on selected parameters of the redox imbalance in infantile and adult animals.

According to our results, neonatal PEG40 administration caused a significant decrease of antioxidant capacity in infantile female rats when compared to controls. If we speculate that TEAC reduction is a result of the elevated oxidative stress after the PEG-*b*-PLA NP administration, we can expect elevated activities of catalase (CAT), GPx, and SOD. Indeed, we have recorded the trend of increased activities of all three enzymes after neonatal PEG20 administration. This elevation was significant after PEG40 administration. Increase of antioxidant enzyme activities such as SOD, GPx, and CAT may be an indicator of the compensation of the induced oxidative stress [30].

Compared to infantile animals, adult female rats are expected to have elevated levels of free radicals and associated decrease in antioxidant capacity of the organism as reported by Haser and Fürll [31]. However, in our study, we have observed no change in TEAC.

SOD, GPx, and CAT belong to the most important antioxidant defense systems in all cells [32]. Several studies have reported the significant age/related increase of free radicals and decline in activities of these antioxidant enzymes in the organism [33, 34]. Despite this fact, we have found increased levels of GPx and SOD in control groups of adult rats when compared to infantile animals (*p* < 0.05). Our results are consistent with the report by Haser and Fürll [31], who found an age-related elevation in activities of antioxidant enzymes but only a nonsignificant change of TEAC in healthy calves.

A downregulation of antioxidative mechanisms as well as an increased production of ROS might be important factors for an age-related increase of oxidative damages [35]. Remarkably, when compared to the control group, significant decrease of GPx and SOD activities in adult rats has been recorded after neonatal administration of PEG40. Moreover, significant decrease in SOD activity has also been monitored after neonatal administration of the lower dose of PEG-*b*-PLA (PEG20). This fact is consistent with the results of Fernandez-Urrusuno et al. [36] who also reported that administration of polymeric NPs induced a depletion of reduced glutathione and an increase of oxidized glutathione levels in the liver, as well as inhibition of SOD activity. Moreover, according to Komosinska-Vassev et al. and Seven et al. [37, 38], reduced activities of antioxidant enzymes may be caused by their increased utilization in protection against oxidative damage, or on the other hand, by the decrease of reactive species formation.

In infantile rats, unchanged levels of protein carbonyls and lipid peroxides after neonatal PEG*-b*-PLA administration could be then related to increased activities of mentioned antioxidant enzymes and to the ability of the organism to cope with the oxidative stress formed by the PEG-*b*-PLA administration. Similarly, in adult rats, the level of lipoperoxides was unchanged, even though we have found decreased levels of protein carbonyls after neonatal PEG20 administration. This decrease and subsequent increase in the original concentration might suggest the stimulation of the antioxidant system at the lower doses of PEG-*b*-PLA NPs. Oxidative stress formed by PEG40 administration cannot be buffered by the antioxidant defense system of the adult organism equally as in infantile rats, which could be the cause of elevated damage to proteins.

In addition, in our study, only female rats were included. We have confirmed the statement of Lutosławska et al. [39] who found the protective effect of estrogens in adult females against lipid peroxidation and oxidative damage to DNA. In control groups, we have detected significantly lower levels (*p* < 0.05) of lipid peroxides in female adult rats when compared to infantile animals.

## 5. Conclusion

As the production and use of NPs have proceeded, humans are predicted to be more exposed to the nanomaterials. Due to their unique properties, NPs have the great potential in the field of nanomedicine. Since the harmful effects of certain NPs were established, understanding the interaction of NPs with biological systems is essential for the development of safe NPs used in biomedicine. In our study, obtained data indicate a possible age-related association between the oxidative stress and neonatal PEG-*b*-PLA administration. Effects of NP used in our study can be summarized as follows:

- Administration of the lower dose of PEG nanoparticles (PEG20) to infantile rats had no effect on measured parameters except catalase activity which was significantly increased compared to controls. In adult animals, the same dose of NP caused the significant reduction of protein carbonyl levels as well as SOD activity.
- Administration of the higher dose of PEG nanoparticles (PEG40) to infantile rats induced a significant reduction in TEAC levels and the elevation of catalase and SOD activities compared to controls. In adult animals, the same dose of NP caused the reduction of antioxidant enzymes activities (GPx and SOD).
- Our results indicate that in infantile rats PEG40 nanoparticles induce an opposite effect on antioxidant enzyme activities than in adults. Antioxidant enzyme activities in infantile rats are significantly reduced while in adults they are significantly elevated.
- There was no effect of PEG40 nanoparticles on the markers of oxidative damage to proteins (protein carbonyls) and lipids (lipoperoxides).

Therefore, it is obvious that further experiments are necessary to clarify the impact of NPs on the whole organism and the mechanism of their action.

## Figures and Tables

**Table 1:** Physicochemical characteristics of PEG-*b*-PLA suspension.

Size (nm)		Zeta potential (mV)
TEM	DLS	
~50	64.9 ± 10.5	28.73 ± 1.44
	911.4 ± 117.6	

TEM: transmission electron micrography; DLS: dynamic light scattering.

**Table 2:** Effects of neonatal PEG-*b*-PLA NP exposure on oxidative status of infantile and adult female Wistar rats.

Parameter	Control	PEG20	PEG40
*Infantile rats*			
TEAC (mmol trolox/l)	3.046 (2.882–3.313)	3.07 (2.952–3.39)	2.124^∗∗##^ (1.826–2.870)
Protein carbonyls (nmol/mg protein)	1.772 (1.43–2.10)	2.305 (2.00–2.356)	1.931 (1.844–2.059)
Lipoperoxides (nmol/ml)	184.861 (154.960–285.039)	222.400 (170.020–251.429)	254.702 (189.663–275.109)
CAT (*μ*kat/g Hb)	0.724 (0.565–0.896)	0.818^∗^ (0.768–1.034)	1.111^∗^ (0.821–1.168)
GPx (nU/ml/mg Hb)	36.585 (31.610–42.125)	43.506 (32.127–49.125)	47.829^∗∗∗^ (45.864–54.859)
SOD (U/mg Hb)	8.347 (6.508–10.736)	11.417 (9.837–12.721)	12.985^∗∗∗^ (11.345–14.144)
*Adult rats*			
TEAC (mmol/l)	2.842 (2.758–3.004)	2.586 (2.518–2.890)	2.951 (2.9525–2.955)
Protein carbonyls (nmol/mg protein)	1.843 (1.799–2.310)	1.692^∗^ (1.649–1.722)	1.863^#^ (1.859–2.087)
Lipoperoxides (nmol/ml)	44.306^++^ (36.230–55.655)	43.541 (34.920–72.788)	46.488 (32.956–53.909)
CAT (*μ*kat/g Hb)	56.538^+++^ (56.078–57.434)	0.592 (0.493–0.633)	0.538 (0.523–0.657)
GPx (nU/ml/mg Hb)	56.538^+++^ (56.078–57.434)	53.204 (46.612–57.434)	41.122^∗^ (40.200–50.426)
SOD (U/mg Hb)	12.479^+^ (10.703–14.847)	8.738^∗^ (7.340–10.202)	6.325^∗^ (4.944–7.938)

TEAC: trolox equivalent antioxidant capacity; CAT: catalase activity; GPx: glutathione peroxidase; SOD: superoxide dismutase activity; PEG20: animals treated with PEG-*b*-PLA NPs (20 mg/kg body weight); PEG40: animals treated with PEG-*b*- PLA NPs (40 mg/kg body weight). Data are presented as the median with interquartile range (Q1-Q3, 25–75%). Statistical analysis was performed using Stats Direct 3 Statistical software and Mann–Whitney *U* test. ^∗^*p* < 0.05 PEG 20 or PEG40 versus control; ^∗∗^*p* < 0.01 PEG 20 or PEG40 versus control; ^∗∗∗^*p* < 0.001 PEG 20 or PEG40 versus control; ^#^*p* < 0.05 PEG40 versus PEG20; ^##^*p* < 0.01 PEG40 versus PEG20; ^+^*p* < 0.05 control-adult rats versus control-infantile rats; ^++^*p* < 0.01 control-adult rats versus control-infantile rats; and ^+++^*p* < 0.001 control-adult rats versus control-infantile rats.

**Table 3:** Significant correlations between measured parameters of infantile and adult rats administered with PEG20, PEG40, or deionized water (controls).

			*r*	*p*
*Infantile rats*				
Controls	Cat	Lpx	0.709	0.0367
PEG20	Cat	GPx	0.818	0.0019
	SOD	Cat	0.903	0.0004
		GPx	0.648	0.0245
		Lpx	−0.551	0.0481
PEG40	GPx	Protein carbonyls	0.648	0.0216
	SOD	Lpx	−0.673	0.0173
*Adult rats*				
PEG20	SOD	Catalase	0.943	0.0083
